# Impact of the COVID-19 Pandemic on Cancer Diagnoses in General and Specialized Practices in Germany

**DOI:** 10.3390/cancers13030408

**Published:** 2021-01-22

**Authors:** Louis Jacob, Sven H. Loosen, Matthias Kalder, Tom Luedde, Christoph Roderburg, Karel Kostev

**Affiliations:** 1 Research and Development Unit, Parc Sanitari Sant Joan de Déu, CIBERSAM, Dr. Antoni Pujadas, 42, Sant Boi de Llobregat, 08830 Barcelona, Spain; louis.jacob.contacts@gmail.com; 2 Faculty of Medicine, University of Versailles Saint-Quentin-en-Yvelines, 78180 Montigny-le-Bretonneux, France; 3 Clinic for Gastroenterology, Hepatology and Infectious Diseases, University Hospital Düsseldorf, Medical Faculty of Heinrich Heine University Düsseldorf, Moorenstraße 5, 40225 Düsseldorf, Germany; Sven.Loosen@med.uni-duesseldorf.de (S.H.L.); tom.luedde@med.uni-duesseldorf.de (T.L.); 4 Department of Gynecology and Obstetrics, Philipps University of Marburg, 35037 Marburg, Germany; kalder@med.uni-marburg.de; 5 Epidemiology, IQVIA, 60549 Frankfurt, Germany; karel.kostev@iqvia.com

**Keywords:** COVID-19 pandemic, cancer diagnosis, general practices, specialized practices, Germany

## Abstract

**Simple Summary:**

Coronavirus disease 2019 (COVID-19) represents a major challenge for global healthcare systems. Since these healthcare systems have been frequently overwhelmed by the large number of COVID-19 patients, it is conceivable to hypothesize that other diseases such as cancer have been neglected during the pandemic. This study showed that the number of new cancer diagnoses in Germany significantly decreased between March and May 2020 compared with 2019. Given that a sudden decline in the actual incidence of cancer is unlikely, these data suggest that a large proportion of cancer cases have been undiagnosed or diagnosed with some delay in this country, and this may be associated with poor short-term and long-term outcomes. Thus, the present study provides important evidence for the vivid discussion on how healthcare systems should optimally deal with the ongoing COVID-19 pandemic.

**Abstract:**

The aim of this retrospective study was to investigate the impact of the coronavirus disease 2019 (COVID-19) pandemic on cancer diagnosis in general and specialized practices in Germany. This study included a total of 102,009 patients aged ≥18 years newly diagnosed with cancer in 1660 practices in Germany from January to May 2019 and from January to May 2020. Practices included general, gynecology, ear, nose, and throat (ENT), dermatology, and urology practices. New cancer diagnoses included all types of cancer and corresponded to cancers not previously documented in the database for a given patient. The number of new cancer diagnoses per general practice decreased significantly between March and May 2020 compared with the same period in 2019 (March: −12.0%, April: −27.6%, and May: −23.4%). A similar trend was observed in specialized practices, and this trend was more pronounced in April 2020 (dermatology: −44.4%, gynecology: −32.0%, and ENT: −28.2%). In addition, there was a significant decrease in almost all sex and age groups in April and May 2020 compared with the same period in 2019. Finally, the decrease in the number of new cancer diagnoses was particularly pronounced among cancers of the skin and the respiratory and intrathoracic organs. Together, these data show that the COVID-19 pandemic had a significant negative impact on cancer diagnosis in Germany, highlighting the need for public health measures improving the management of cancer in this country during this ongoing pandemic.

## 1. Introduction

Coronavirus disease 2019 (COVID-19) represents a major challenge for global healthcare systems [1]. As of 21 December 2020, there have been 75,479,471 confirmed cases of COVID-19 with 1,686,267 related deaths globally [2]. COVID-19 is caused by severe acute respiratory syndrome coronavirus-2 (SARS-CoV-2) and involves pulmonary (e.g., rhinorrhea, cough, and dyspnea) as well as extra-pulmonary symptoms (e.g., anosmia, dizziness, and nausea/vomiting) [3,4]. 

Outpatient care in Germany is formed by practices of general practitioners and specialists [5]. Patients are usually referred by their general practitioners to specialized practices, and this referral takes less than a few weeks for urgent cases. Both types of practices are involved in the management of cancer and particularly in German cancer screening programs. These voluntary programs, which adults insured under statutory health insurance (GKV) are entitled to, include screening for skin cancer by examination of the skin every two years, colorectal cancer screening by colonoscopy every 10 years starting at the age of 55 years (or by occult blood testing if patients refuse colonoscopy), screening for cervix cancer by swab test on a yearly base starting at the age of 20 years, mammography for breast cancer starting at age of 50 years, and screening for prostate cancer on a yearly base starting at the age of 45 years by palpation [6]. To reduce the risk of an overload of the healthcare system as it had been observed in several countries around the world [7,8], the German Federal Government took drastic measures in the beginning of 2020 [9]. These measures included the postponement of non-urgent clinical visits, the cancellation of elective surgeries, an increase in intensive care capacities, and the restriction of social life with a national lockdown. Although these measures are effective in reducing the number of new COVID-19 infections [10], they may also have a deleterious impact on the diagnosis of other major medical conditions. For example, previous research has indicated that the rate of stroke [11], acute heart failure [12], and pulmonary embolism diagnoses [13] has fallen during the COVID-19 pandemic, which may be explained by a decrease in the proportion of symptomatic patients seeking medical care. Several studies have also shown that the incidence of cancer has decreased during lockdown in a number of countries [14,15,16,17,18,19,20,21]. Some cancer diagnoses might have been more affected by the COVID-19 than other cancer diagnoses, and cancers detected in the context of screening programs might have been particularly impacted. As a matter of fact, a study from a secondary care hospital in Italy revealed that the incidence of cancer diagnosis decreased by 39% in 2020 with prostate (75%), bladder (66%), and colorectal cancer (62%) being the most impacted tumor entities [14]. In another study from the United Kingdom, it was further observed that the prevalence of cancer referrals (−34%) and skin cancer diagnoses (−53%) decreased between February and April 2020 compared with 2019 [16]. Interestingly, prostate, colorectal, and skin cancers are often diagnosed in patients undergoing specific cancer screening programs. Importantly, for the majority of cancers, curative treatment is only feasible when the tumor is diagnosed at an early stage. A delay in the diagnosis of cancer due to the COVID-19 pandemic might thus result in an increase in cancer-related mortality [22]. Notwithstanding the fact that this body of literature has yielded some important findings regarding the relationship between the COVID-19 pandemic and cancer diagnoses, these studies are subject to a number of limitations that need to be acknowledged. First, some of the studies were conducted in single centers [14,16,18], which may undermine the generalizability of their findings. Second, not all types of cancer were included in the analyses [16,18,20], and decreases in the rate of diagnosis may vary with the type of cancer. Taking these limitations into consideration, more data from a variety of settings are needed to better estimate the change in cancer diagnosis rates during this global health crisis. 

Therefore, the goal of this retrospective study was to investigate the impact of the COVID-19 pandemic on cancer diagnosis in general and specialized practices in Germany. To the best of our knowledge, no research has yet been performed analyzing the effects of the COVID-19 pandemic and the related lockdown on cancer diagnosis in this country (first day of lockdown: 23 March 2020 [23]).

## 2. Materials and Methods

### 2.1. Database

This study used data from the Disease Analyzer database (IQVIA). Full details of the database have been published elsewhere [24]. Briefly, the sampling method for the Disease Analyzer database is based on summary statistics from all doctors in Germany published yearly by the German Medical Association. The panel design of the database is determined using several strata (i.e., age of physician, specialist group, community size category, and German federal state). Data are encrypted for data protection before transmission, and sociodemographic, diagnosis and prescription data from general and specialized practices are transmitted to IQVIA on a monthly basis. Diagnosis data are based on the German adaptation of the International Classification of Diseases, 10th revision (ICD-10). Given that the Disease Analyzer database does not provide information on patients’ specific symptoms, new cancer diagnoses correspond to cancers that are documented for the first time in the database by physicians with the ICD-10 classification. The quality of the data is assessed regularly by IQVIA based on a number of criteria (e.g., completeness of documentation and linkage between diagnoses and prescriptions). Finally, it has previously been shown that the panel of practices included in the Disease Analyzer database is representative of general and specialized practices in Germany [24].

### 2.2. Study Population and Variables

This retrospective study included all patients aged ≥18 years with at least one visit to one of 1660 practices in Germany between January and May 2019 and January and May 2020 (*n* = 4,770,468). Practices included general, gynecology, ear, nose, and throat (ENT), dermatology, and urology practices. New cancer diagnoses included all types of cancer (ICD-10: C00-C97). Only patients who had never been diagnosed with any kind of cancer in their entire medical history were included in this study. New cancer diagnoses were also studied by cancer site: skin (ICD-10: C43 and C44; dermatology and general practices); male genital organs including prostate (ICD-10: C60-C63; urology practices); urinary tract (ICD-10: C64-C68; urology practices); breast (ICD-10: C50; gynecology practices); lip, oral cavity, and pharynx (ICD-10: C00-C14; ENT practices); female genital organs (ICD-10: C50-C59; gynecology practices); digestive organs (ICD-10: C15-C26; general practices); respiratory and intrathoracic organs (ICD-10: C30-C39; ENT and general practices); lymphoid, hematopoietic, and related tissue (ICD-10: C81-C97; general practices); thyroid and other endocrine glands (ICD-10: C73-C75; general practices); and head/brain (ICD-10: C69-C72; general practices). Finally, sociodemographic variables included sex and age. 

### 2.3. Statistical Analyses

The number of new cancer diagnoses each month per practice for the period between January and May was compared for 2019 and 2020 using Wilcoxon tests. Comparisons were stratified by practice type, sociodemographic characteristics (sex and age), and cancer site. A *p*-value of <0.05 was considered statistically significant. The analyses were carried out using SAS 9.4. 

## 3. Results

### 3.1. Patients’ and Practices’ Characteristics

In order to investigate the potential impact of the COVID-19 pandemic on the diagnosis of cancer in Germany, this study compared the number of new cancer diagnoses in 1660 general and specialized practices in Germany between January and May 2019 and January and May 2020 (see Materials and Methods for details). A total of 54,867 patients newly diagnosed with cancer between January and May 2019 and 47,142 patients newly diagnosed with cancer between January and May 2020 were included in this study. Mean age of the study population was 64.4 (SD = 16.2) years in 2019 and 64.7 (SD = 16.2) years in 2020. The prevalence of women was 52.0% and 50.4% in 2019 and 2020, respectively. In terms of practices, there were 1090 general, 242 gynecology, 146 ENT, 97 dermatology, and 85 urology practices. 

### 3.2. Decreased Number of New Cancer Diagnoses during the COVID-19 Pandemic

Hypothesizing that the 2020 COVID-19 pandemic might negatively influence the diagnosis of cancer in Germany, the mean number of new cancer diagnoses per practice was compared between January and May 2019 and the same period in 2020. Interestingly, while the number of new cancer diagnoses per general practice was comparable between January and February 2019 and January and February 2020, it was observed that the number of new cancer diagnoses per general practice significantly decreased in March, April, and May 2020 compared with the respective months in 2019 (March: −12.0%, April: −27.6%, and May: −23.4%; Table 1). This trend was also observed in specialized practices. Indeed, there was a 30% decrease in the number of new cancer diagnoses per gynecological practice in April and May 2020 compared with the same period in 2019 (April: −32.0% and May: −30.8%). Similarly, the number of new cancer diagnoses fell during the COVID-19 pandemic in ENT and dermatology practices, and this decrease was particularly pronounced in April (ENT: −28.2% and dermatology: −44.4%). Finally, the number of new cancer diagnoses also decreased in urology practices in April and May 2020 compared with April and May 2019, but statistical significance was not reached.

### 3.3. Age and Sex Differences in the Decrease in the Number of New Cancer Diagnoses per Practice during the COVID-19 Pandemic

Age- and sex-stratified analyses were further conducted to assess potential age and sex differences in the impact of COVID-19 on the number of new cancer diagnoses per practice (Table 2). A significant decrease in the number of new cancer diagnoses from April–May 2020 compared with the same period in 2019 was observed in almost all age and sex subgroups. Interestingly, this decrease was particularly pronounced in certain demographic subgroups. For example, the number of new cancer diagnoses decreased in April 2020 by 38.7% in women aged 71–80 years, 36.0% in those aged 18–40 years, and 33.3% in those aged > 80 years. On the contrary, this decrease was not statistically significant in men aged 18–40 years in April 2020 compared with April 2019 (−7.9%).

### 3.4. Differences by Cancer Site in the Decrease in the Number of New Cancer Diagnoses per Practice during the COVID-19 Pandemic

The final aim of this study was to identify potential differences by cancer site in the decrease in the number of new cancer diagnoses per practice (Table 3). Interestingly, the decrease in the number of new cancer diagnoses during the COVID-19 pandemic varied with cancer site. The diagnosis of skin cancers was the most impacted by the COVID-19 pandemic in both dermatology (March: −25.6% and April: −42.9%) and general practices (March: −19.6% and April: −29.3%). In addition, the number of new diagnoses of respiratory and intrathoracic organ cancers in ENT practices decreased by 19.9% in March and by 40.0% in April 2020. By contrast, this decrease was relatively low for other cancers, such as digestive organ cancers or cancers of the thyroid and other endocrine glands, while there was even a non-significant increase in the number of head/brain cancer diagnoses from March–May 2020 compared with the same period in 2019.

## 4. Discussion

### 4.1. Main Findings

There was a significant decrease in the number of new cancer diagnoses in general practices between March and May 2020 compared with the same period in 2019. A similar trend was observed in specialized practices (dermatology, gynecology, and ENT), and this trend was more pronounced in April 2020. Interestingly, the decrease in the number of new cancer diagnoses was significant in almost all sex and age groups with the exception of men aged 18–40 years. Finally, the skin and the respiratory and intrathoracic organs were the two cancer sites that were most significantly impacted by the COVID-19 pandemic. To the best of our knowledge, this is the first study to have investigated the effects of the COVID-19 pandemic and the related lockdown on cancer diagnosis in Germany.

### 4.2. Interpretation of the Findings

The findings of this retrospective study are in line with the literature. For example, a study using data from the national Dutch Cancer Registry showed a decrease of 26–60% in the number of new cancer diagnoses between 6 April 2020 and 12 April 2020, less than a month after the implementation of strict social distancing policies in the Netherlands [15]. Another cross-sectional study of 278,778 patients from the United States revealed that the weekly number of new cancer diagnoses decreased by 46.4% during the COVID-19 pandemic, with this decrease ranging from 24.7% for pancreatic cancer to 51.8% for breast cancer [17]. Finally, an analysis conducted in the United Kingdom found an 88% decrease in the weekly number of endoscopy procedures between March and May 2020 compared with the period between January and March 2020, while the number of weekly cancer diagnoses also decreased by 58% [20].

There are at least two hypotheses that may explain the fall in the number of new cancer diagnoses during the COVID-19 pandemic in Germany and in other countries. First, patients with symptoms (e.g., skin lesion, breast lump, or stomach pain) may not seek medical care because of a fear of contracting COVID-19 [25]. Interestingly, an Italian study showed that patients with a suspected breast lesion and those with diagnosed breast cancer frequently refused surgery because of the risk of developing symptoms of COVID-19 [26]. Furthermore, one retrospective analysis of data from three Dutch hospitals showed that the lockdown in the Netherlands was preceded by decreased emergency department utilization [27], suggesting that fear may play a significant role in decreasing rates of healthcare utilization. In addition, semi-structured interviews conducted in the United States revealed that patients frequently see hospitals as infectious reservoirs [28] and may therefore be reluctant to go to hospital or to make an appointment with a primary care physician. Second, because of the high number of patients infected with COVID-19, healthcare systems were rapidly overloaded and non-urgent care services in hospital and primary care settings were delayed or suspended. A qualitative interview study of 132 general practitioners from Belgium indicated that telephone triage and telephone consultations were favored following the COVID-19 outbreak, while chronic care was usually postponed [29]. A study of 970 dermatologists from the United States further showed a decrease in the number of patients seen per practice (63.4 vs. 149.4), the number of practice days (3.1 vs. 4.2), and the number of biopsies performed for suspicious pigmented skin lesions (7.7 vs. 19.8) in the week of 16 March 2020 compared with the week of 17 February 2020, while nonessential appointments were more frequently postponed (79.4% vs. 35.5%) [30]. Finally, although we did not find a significant decrease in the number of new cancer diagnoses in urology practices, previous research including 766 urologists living in Brazil found a significant decline in the number of patient visits and a high proportion of delayed surgeries [31]. The lack of a significant result in the present study conducted in Germany may be explained by a lack of statistical power and a relatively small number of urology practices available for the analyses. These factors might also represent a possible explanation for the non-significant trend towards a decreasing number of cancer diagnoses that we observed in general, gynecology, ENT, and dermatology practices as early as in February 2020. It should be noted, however, that the COVID-19 pandemic in Germany had not yet received a great deal of public attention in February 2020. Overall, taken together, these findings underline the fact that delays in cancer diagnosis may be particularly significant during the COVID-19 pandemic, and this increased risk of late diagnosis results from both patient-related (i.e., symptomatic patients not seeking medical care) and health system-related delay (i.e., consultations and diagnostic tests being postponed).

The deleterious effects of delays in cancer diagnosis and treatment on prognosis may vary with the type of cancer [32]. For example, the literature investigating the association between delay and prognosis in patients with breast cancer has yielded conflicting results [33]. Whilst longer delays in breast cancer diagnosis were identified as a risk factor for poorer survival rates in a meta-analysis of 87 studies published largely before 1970 [34], several more recent studies did not find any significant relationship between delay and survival [35,36,37]. Therefore, routine mammography screening might be safely delayed in the general population, while further recommendations on breast cancer diagnosis should be established for high-risk individuals [38]. By contrast, in the case of pancreatic cancer [39] and multiple myeloma [40], late diagnosis is a well-known risk factor for increased mortality rates. Interestingly, a modeling study has analyzed the effects of different lockdown scenarios and delayed cancer referrals during the COVID-19 pandemic on cancer survival and found that the different scenarios would result in a considerable number of deaths and life-years lost [41].

### 4.3. Implications and Directions for Future Research

Based on the results of this retrospective study, it can be concluded that the diagnosis of cancer was suboptimal in primary care practices in Germany between March and May 2020 during the COVID-19 pandemic. Given that the COVID-19 pandemic is still ongoing in Europe [2], measures should be taken to improve cancer diagnosis in this context of limited healthcare resources. First, more accessible educational material is needed to improve the understanding of the general population with regard to the COVID-19 pandemic and increase awareness of the fact that the risk of contamination is relatively low when all effective protective measures are respected [42]. Second, patients should also be better informed about symptoms requiring medical care without delay (e.g., recent asymmetric skin lesion or rectal bleeding). Third, an increased number of teleconsultations with primary care physicians is warranted, and patients should be referred promptly to specialists when further exploration is needed. In terms of future research, further studies should focus on the factors that have led to a decrease in the number of new cancer diagnoses in several countries during the COVID-19 pandemic (e.g., patient and health system delay), which should lead to better comprehension of the exact role played by these different factors.

### 4.4. Strengths and Limitations

The major strengths of this study are the number of practices included in the analyses and the use of data that are representative of general and specialized practices in Germany. Nevertheless, the study results should be interpreted in the light of several limitations. First, cancer diagnoses were documented using ICD-10 codes only, and no information was available on the clinical context in which the diagnoses were made or the length of time between the initial onset of symptoms/first consultation and the actual diagnosis. Second, patients diagnosed with cancer in general practices may have been referred to specialized practices and hospitals, but these data were not available in the Disease Analyzer database. Third, it is possible that a significant proportion of patients newly diagnosed with cancer consulted emergency departments directly. Unfortunately, no data in this regard were available from hospitals, and the number of new cancer diagnoses may therefore have been underestimated. Fourth, absolute numbers of cancer diagnoses per practice were relatively small, and this could explain why some differences were not significant in specific groups (e.g., in men aged 18–40 years in April or in women aged 18–40 years in May).

## 5. Conclusions

Overall, the COVID-19 pandemic was associated with a decrease in the number of new cancer diagnoses in German general and specialized practices between March and May 2020. As the COVID-2019 pandemic is still ongoing in Europe, measures should rapidly be taken to improve cancer diagnosis in this context of limited healthcare resources. Finally, further research is needed to gain a better understanding of the factors that have led to a fall in the number of patients newly diagnosed with cancer.

## Figures and Tables

**Table 1:** Differences by practice type in the number of new cancer diagnoses per practice in Germany between January and May 2020 and January and May 2019.

Type of Practice	2019					2020					Difference between 2020 and 2019 (%)				
	Jan	Feb	Mar	Apr	May	Jan	Feb	Mar	Apr	May	Jan	Feb	Mar	Apr	May
General (*n* = 1090)	4.3 (4.5)	3.7 (4.0)	3.9 (4.7)	4.0 (4.5)	3.9 (4.2)	4.4 (4.8)	3.5 (4.1)	3.4 (4.0)	2.9 (3.1)	3.0 (3.3)	4.0	−6.2	**−12.0**	**−27.6**	**−23.4**
Gynecology (*n* = 242)	4.9 (8.4)	4.1 (6.4)	4.5 (6.9)	4.0 (5.8)	4.4 (7.1)	4.5 (5.2)	3.7 (4.5)	3.5 (4.1)	2.7 (2.7)	3.0 (3.9)	−8.1	−9.7	−21.7	**−32.0**	**−30.8**
ENT (*n* = 146)	4.1 (4.5)	3.5 (4.3)	3.4 (4.6)	3.7 (4.0)	3.9 (4.4)	4.3 (4.3)	3.3 (3.3)	2.9 (3.2)	2.6 (2.7)	3.3 (3.2)	6.0	−4.9	−15.0	**−28.2**	−15.5
Dermatology (*n* = 97)	37.6 (50.3)	35.0 (39.1)	36.3 (52.9)	34.7 (55.2)	35.6 (50.2)	37.9 (54.3)	33.7 (44.3)	28.0 (28.3)	19.3 (24.6)	28.0 (36.1)	0.9	−3.6	−23.0	**−44.4**	−21.3
Urology (*n* = 85)	20.1 (25.2)	19.1 (26.4)	19.1 (26.9)	17.8 (23.9)	19.7 (27.3)	21.0 (27.5)	19.3 (26.9)	20.0 (27.4)	14.5 (19.3)	16.8 (23.3)	4.9	0.9	4.7	−18.1	−14.8

Abbreviation: ENT, ear, nose, and throat. Significant differences are displayed in bold (*p*-value < 0.05). Data shown are mean (standard deviation) unless otherwise indicated.

**Table 2:** Differences by sex and age in the number of new cancer diagnoses per practice in Germany between January and May 2020 and January and May 2019.

Sex	Age Group	2019					2020					Difference between 2020 and 2019 (%)				
		Jan	Feb	Mar	Apr	May	Jan	Feb	Mar	Apr	May	Jan	Feb	Mar	Apr	May
Male	18–40 years	0.3 (1.4)	0.3 (1.2)	0.3 (1.2)	0.3 (1.4)	0.3 (1.5)	0.3 (1.9)	0.3 (1.4)	0.3 (1.6)	0.2 (1.3)	0.2 (1.3)	15.5	7.3	17.0	−7.9	**−17.7**
	41–50 years	0.4 (1.2)	0.3 (1.2)	0.3 (1.2)	0.3 (1.0)	0.3 (1.4)	0.3 (1.5)	0.3 (1.3)	0.3 (1.1)	0.2 (0.9)	0.2 (1.0)	−6.2	−5.7	**−13.6**	**−22.9**	**−27.9**
	51–60 years	0.8 (2.3)	0.7 (2.2)	0.8 (2.4)	0.7 (2.1)	0.7 (2.4)	0.9 (3.1)	0.8 (2.8)	0.8 (2.8)	0.6 (2.3)	0.7 (2.4)	10.3	7.5	4.4	**−19.5**	−11.0
	61–70 years	1.0 (2.6)	0.9 (2.4)	1.0 (2.7)	0.9 (2.3)	1.0 (2.5)	1.1 (2.8)	0.9 (2.3)	0.9 (2.4)	0.7 (1.6)	0.8 (2.3)	3.4	−3.2	−7.4	**−23.5**	**−17.6**
	71–80 years	1.1 (2.3)	1.1 (2.4)	1.0 (2.5)	1.1 (2.3)	1.0 (2.3)	1.2 (2.6)	0.9 (2.3)	0.9 (1.9)	0.7 (1.5)	0.8 (1.9)	7.6	**−11.3**	−15.2	**−32.4**	**−21.5**
	>80 years	0.7 (1.5)	0.6 (1.4)	0.6 (1.4)	0.6 (1.4)	0.7 (1.5)	0.8 (1.5)	0.6 (1.3)	0.6 (1.2)	0.5 (1.0)	0.6 (1.2)	6.8	−5.8	−6.9	**−21.0**	**−15.0**
Female	18–40 years	0.5 (2.4)	0.4 (1.9)	0.4 (2.5)	0.4 (2.9)	0.4 (2.4)	0.5 (2.5)	0.4 (1.9)	0.4 (1.9)	0.3 (1.6)	0.4 (2.2)	−4.4	−6.7	−18.9	**−36.0**	−15.2
	41–50 years	0.4 (1.7)	0.4 (1.4)	0.4 (1.5)	0.4 (1.6)	0.4 (1.5)	0.4 (1.5)	0.3 (1.4)	0.4 (1.2)	0.3 (1.1)	0.3 (1.2)	−5.8	−13.8	−16.1	**−26.3**	**−25.9**
	51–60 years	0.8 (2.4)	0.7 (2.2)	0.8 (2.7)	0.8 (2.2)	0.7 (2.4)	0.9 (2.7)	0.8 (2.6)	0.7 (2.2)	0.5 (2.0)	0.6 (2.0)	5.7	5.1	−16.5	**−27.0**	**−18.7**
	61–70 years	0.9 (2.2)	0.8 (2.0)	0.8 (2.3)	0.8 (2.2)	0.8 (2.1)	0.9 (2.4)	0.7 (2.0)	0.7 (1.9)	0.5 (1.3)	0.6 (2.0)	2.5	−2.4	−12.7	**−32.8**	**−25.1**
	71–80 years	0.8 (1.9)	0.7 (1.8)	0.7 (1.7)	0.8 (1.8)	0.8 (1.8)	0.8 (1.9)	0.7 (1.7)	0.6 (1.5)	0.5 (1.0)	0.6 (1.5)	−5.2	−7.9	−13.0	**−38.7**	**−20.5**
	>80 years	0.6 (1.2)	0.5 (1.2)	0.6 (1.3)	0.6 (1.1)	0.6 (1.2)	0.7 (1.5)	0.6 (1.3)	0.5 (1.0)	0.4 (0.8)	0.5 (1.0)	**22.2**	2.9	**−19.0**	**−33.3**	**−16.6**

Significant differences are displayed in bold (*p*-value < 0.05). Data shown are mean (standard deviation) unless otherwise indicated.

**Table 3:** Differences by cancer site in the number of new cancer diagnoses per practice in Germany between January and May 2020 and January and May 2019.

Cancer Site	2019					2020					Difference between 2020 and 2019 (%)				
	Jan	Feb	Mar	Apr	May	Jan	Feb	Mar	Apr	May	Jan	Feb	Mar	Apr	May
Skin (dermatology)	33.3 (49.7)	30.9 (37.6)	32.4 (51.6)	30.4 (53.9)	31.5 (49.5)	33.8 (52.8)	30.1 (41.9)	24.1 (26.3)	17.3 (23.8)	24.6 (34.8)	1.8	−2.4	**−25.6**	**−42.9**	−22.0
Male genital organs including prostate (urology)	11.0 (13.4)	10.7 (15.5)	10.3 (14.7)	9.6 (12.7)	11.2 (15.1)	12.1 (17.4)	10.9 (16.7)	11.5 (15.6)	8.3 (10.6)	10.3 (15.1)	10.5	1.8	12.0	−13.1	−8.1
Urinary tract (urology)	6.4 (18.7)	6.5 (20.0)	6.9 (20.5)	6.5 (18.5)	7.0 (21.0)	6.8 (20.3)	7.1 (20.5)	6.9 (20.7)	5.6 (13.8)	6.0 (17.5)	5.5	8.6	1.2	−14.5	−13.4
Breast (gynecology)	3.4 (5.9)	2.8 (4.4)	2.9 (5.0)	2.9 (4.3)	2.8 (4.6)	2.8 (3.2)	2.5 (2.0)	2.4 (2.1)	2.1 (1.6)	2.3 (2.7)	−16.1	−12.4	−17.0	−24.9	−16.2
Lip, oral cavity, and pharynx (ENT)	2.4 (3.9)	2.2 (3.8)	2.5 (4.5)	1.9 (2.5)	1.7 (2.4)	2.1 (3.1)	1.8 (2.1)	1.8 (1.7)	1.8 (2.2)	1.5 (0.9)	−10.5	−19.3	−30.4	−2.0	−12.1
Skin (general)	2.3 (3.3)	2.1 (2.8)	2.1 (3.6)	2.2 (3.1)	2.1 (3.2)	2.1 (2.8)	1.9 (2.9)	1.7 (1.9)	1.5 (1.4)	1.8 (1.8)	−4.1	**−9.2**	**−19.6**	**−29.3**	−17.2
Female genital organs (gynecology)	1.9 (3.0)	2.3 (2.9)	1.9 (2.4)	1.8 (2.0)	2.4 (4.1)	2.0 (3.4)	2.0 (3.6)	1.8 (2.6)	1.5 (1.3)	1.7 (1.5)	5.3	−16.3	−3.9	−15.9	−26.4
Digestive organs (general)	1.7 (2.1)	1.5 (1.5)	1.6 (1.9)	1.5 (1.0)	1.6 (1.7)	1.7 (1.5)	1.6 (1.3)	1.5 (1.1)	1.4 (0.9)	1.5 (1.2)	−2.3	3.5	−3.7	−6.0	−5.6
Respiratory and intrathoracic organs (ENT)	1.6 (1.3)	1.7 (1.8)	1.7 (1.8)	2.0 (1.9)	2.1 (2.8)	1.5 (1.4)	1.6 (1.3)	1.4 (0.7)	1.2 (0.5)	1.6 (1.3)	−6.3	−4.7	**−19.9**	**−40.0**	−22.1
Lymphoid, hematopoietic, and related tissue (general)	1.6 (2.0)	1.5 (2.3)	1.6 (3.2)	1.6 (2.4)	1.5 (2.2)	1.6 (2.1)	1.6 (2.3)	1.5 (1.6)	1.4 (0.8)	1.5 (1.5)	1.9	4.3	−5.6	−12.4	−0.3
Respiratory and intrathoracic organs (general)	1.3 (1.3)	1.3 (0.7)	1.4 (1.2)	1.3 (0.8)	1.3 (0.8)	1.4 (1.0)	1.3 (1.3)	1.4 (1.0)	1.3 (1.0)	1.2 (0.7)	3.4	4.9	2.5	−5.2	−4.6
Thyroid and other endocrine glands (general)	1.1 (0.4)	1.1 (0.3)	1.2 (0.7)	1.2 (0.5)	1.1 (0.4)	1.1 (0.5)	1.2 (0.4)	1.2 (1.2)	1.1 (0.3)	1.1 (0.3)	2.7	**10.0**	−0.2	−7.8	2.2
Head/brain (general)	1.1 (0.4)	1.1 (0.3)	1.1 (0.2)	1.0 (0.2)	1.0 (0.2)	1.1 (0.2)	1.0 (0.2)	1.1 (0.3)	1.1 (0.4)	1.1 (0.3)	−3.4	−4.4	2.9	6.4	4.6

Abbreviation: ENT, ear, nose, and throat. Significant differences are displayed in bold (*p*-value < 0.05). Data shown are mean (standard deviation) unless otherwise indicated.

## Data Availability

The data that support the findings of this study are available on request from the corresponding author.
